# Microstructure of the superior temporal gyrus and hallucination proneness - a multi-compartment diffusion imaging study

**DOI:** 10.1016/j.nicl.2018.06.027

**Published:** 2018-06-25

**Authors:** Amy Spray, Anton L. Beer, Richard P. Bentall, Vanessa Sluming, Georg Meyer

**Affiliations:** a University of Liverpool, Liverpool, UK; b University of Regensburg, Regensburg, Germany; c University of Sheffield, Sheffield, UK

**Keywords:** Hallucination, Superior temporal gyrus, LSHS, Schizophrenia, NODDI, Diffusion MRI, AVH, auditory verbal hallucination, CVH, clinical voice hearer, DTI, diffusion-tensor imaging, DWI, diffusion-weighted imaging, FA, fractional anisotropy, fMRI, functional magnetic resonance imaging, HVH, healthy voice hearer, LSHS, Launey Slade Hallucination Scale, MD, mean diffusivity, MRI, magnetic resonance imaging, NDI, neurite density index, NODDI, neurite orientation dispersion and density imaging, ODI, orientation dispersion index, ROI, region of interest, STG, superior temporal gyrus

## Abstract

Previous studies reported that the volume of the left superior temporal gyrus (STG) is reduced in patients with schizophrenia and negatively correlated with hallucination severity. Moreover, diffusion-tensor imaging studies suggested a relationship between the brain microstructure in the STG of patients and auditory hallucinations. Hallucinations are also experienced in non-patient groups. This study investigated the relationship between hallucination proneness and the brain structure of the STG.

Hallucination proneness was assessed by the Launey Slade Hallucination Scale (LSHS) in 25 healthy individuals who varied in their propensity to hear voices. Brain volume and microstructure of the STG was assessed by magnetic resonance imaging (MRI). Microstructure was examined by conventional diffusion-tensor imaging as well as by neurite orientation dispersion and density imaging (NODDI). The latter decomposes diffusion-based MRI into multiple compartments that characterize the brain microstructure by its neurite complexity known as orientation dispersion (ODI) and by its neurite density (NDI).

Hallucination proneness was negatively correlated with the volume and microstructure (fractional anisotropy, neurite complexity) of the left but not the right STG. The strongest relationship (*r* = −0.563) was observed for neurite complexity (ODI). No correlation was observed for neurite density (NDI).

These findings suggest that there is a relationship between the volume and the microstructure of the left STG and hallucination proneness. Dendritic complexity (but not neurite density) is inversely related to hallucination proneness. Metrics based on multi-compartment diffusion models seem to be more sensitive for hallucination-related neural processes than conventional MRI-based metrics.

## Highlights

• Hallucination proneness is predicted by structural characteristics of left STG
• Hallucination proneness negatively correlates with left STG volume
• Hallucination proneness negatively correlates with left STG fractional anisotropy
• Hallucination proneness negatively correlates with left STG orientation dispersion
• Reduced functional integration may be due to reduced dendritic complexity of neurons

## 1 Introduction

Auditory verbal hallucinations (AVHs), or ‘hearing voices’, in the absence of any external auditory stimulus is the most commonly reported symptom of schizophrenia with a prevalence of around 70% (Sartorius et al., 1986). AVHs are also experienced by a significant minority of the general population (Allen et al., 2012; Beavan et al., 2011; Jardri et al., 2012; Verdoux and van Os, 2002), who are sometimes referred to as healthy voice hearers (HVHs), with an estimated lifetime prevalence of 4% to 15% (Van Os et al., 2009). For this reason, it was proposed that AVHs may represent part of an ‘extended phenotype’ of psychosis (Johns et al., 2004). According to this hypothesis psychotic symptoms are represented along a continuum which includes the (healthy) general population (Baumeister et al., 2017; Bentall, 2004; Daalman et al., 2011). This continuum accommodates, at one end, clinical voice hearers (CVHs) who are distressed by their voices and require care and, at the other end, non-voice-hearing healthy individuals (healthy controls) (Baumeister et al., 2017).

Despite of the large prevalence of AVH in clinical and non-clinical populations their aetiology remained poorly understood. Several functional magnetic resonance imaging (fMRI) studies that investigated the brain activity during AVHs in CVHs identified the contribution of fronto-temporal language circuits, including parts of the auditory cortex (*for review see* (Allen et al., 2008). Several studies specifically implicated the superior temporal gyrus (STG), which is closely associated with speech and language processing (Hickok and Poeppel, 2000) and contains Wernicke's area which is integral for speech perception (*for review see* (Jardri et al., 2012). These findings support the theory that AVH involve the auditory pathways that process speech (Allen et al., 2007). Although the vast majority of research has focused on CVHs, fMRI studies looking at AVHs in non-clinical voice hearers also reported similar activation patters in the left STG (Allen et al., 2012; Daalman et al., 2011; Diederen et al., 2012; Hill and Linden, 2013; Jardri et al., 2012; Linden et al., 2011), supporting the hypothesis that the neural mechanisms behind AVHs are the same in clinical and non-clinical populations (Jardri et al., 2012).

Altered functional activation patterns of AVHs are also accompanied by differences in the brain structure as revealed by conventional magnetic resonance imaging (MRI). In CVHs the most consistently reported structural finding is a reduced volume in the STG of people experiencing hallucinations (Allen et al., 2008; Allen et al., 2012; Jardri et al., 2012). This reduction in volume has also been shown to correlate with AVH severity (Allen et al., 2012; Modinos et al., 2013). A recent review of STG volume-related differences in schizophrenia patients suggests that the left STG is more often implicated than the right hemisphere homologue (Sun et al., 2009). Although this suggests a link between AVH and the STG volume, the cause of this relationship remained unresolved.

Diffusion-weighted imaging (DWI) is an MRI-based method sensitive to the diffusion of water (Le Bihan and Breton, 1985). As this diffusion is constrained by the cellular arrangement, DWI is sensitive to the brain microstructure (Beaulieu, 2002). Conventional DWI methods such as diffusion-tensor imaging (DTI) suggest that AVHs are related to an aberrant microstructure in the STG. For instance, CVHs showed increased diffusivity in the STG compared to healthy people and diffusivity in the left STG was also correlated with symptom severity (Lee et al., 2009). DTI is sensitive to the brain microstructure, but it adopts a relatively simple model (Le Bihan and Breton, 1985). DTI approximates the diffusion at each voxel by an ellipsoid that assumes a single compartment. Hence, it does not well distinguish between different types of cellular assemblies such as neurite structures (e.g., axons and dendrites) or extra-neurite structures (e.g., glia) (Zhang et al., 2012).

Neurite orientation dispersion and density imaging (NODDI) is based on a multi-compartment biophysical model that extends conventional DWI and allows diffusion to be modelled separately for intra-neural and extra-neural space (Le Bihan and Breton, 1985; Zhang et al., 2011; Zhang et al., 2012). Two of the main microstructural markers provided by this method are: the neurite density index (NDI), which estimates the fraction of tissue which is made up of neurites, and the orientation dispersion index (ODI), which estimates the angular configuration of neurites (Zhang et al., 2012). Quantifying neurite morphology in terms of its density and orientation distribution provides further insight into the structural basis of brain function. The branching complexity and orientation of dendritic trees is related to the computational properties and the function of neurons. For instance, neurite morphology is a key characteristic of brain development (Chang et al., 2015; Conel, 1967), aging (Chang et al., 2015; Dickstein et al., 2007) and neurological disorders (Colgan et al., 2016; Dickstein et al., 2007; Zhang et al., 2012). The intra-neurite compartment in grey matter of healthy developed brains is highly dispersed due to sprawling dendritic processes and this would be characterized by high ODI values (Zhang et al., 2012).

Characterising the spatial configuration of neurites in healthy individuals alongside a measure of individual differences in hallucination proneness may therefore offer further insight into the aetiology of AVH. According to the continuum model of AVHs (Baumeister et al., 2017; Van Os et al., 2009; Verdoux and van Os, 2002) both clinical and non-clinical populations should share common mechanisms. By studying non-clinical voice hearers the mechanisms leading to hallucinations may be investigated whilst avoiding confounding effects on the neuroimaging data associated with clinical sequelae such as medication, institutionalization or illness duration.

The present study utilized NODDI measures, alongside conventional structural imaging and DTI, to assess the microstructure in the STG in a non-clinical sample that varied in their propensity to experience hallucinations. Hallucination proneness was assessed using the Launey-Slade Hallucination Scale (LSHS) (Launay and Slade, 1981), which has been widely used in hallucination research, and which is reliable (Bentall and Slade, 1985) and stable over time (Aleman et al., 1999). We hypothesised that volume and parameters defining grey matter microstructure in the STG will be associated with the propensity to hallucinate. In particular we expected a small volume, a low FA value and a low ODI and/or NDI value to be associated with higher scores on the LSHS. As previous research reported a hemisphere bias (Lee et al., 2009; Sun et al., 2009), we expected the association between brain volume, microstructure and LSHS to be most prominent in the left hemisphere.

## 2 Material and methods

### 2.1 Participants

Twenty-five participants aged 20–63 years (M = 39.4, SD = 14.4) were recruited either via an opportunistic sampling method or via an experimental participation programme in the School of Psychology at Liverpool University, in which case they were awarded course credits for their participation, 16 of them were women. 19 participants were students (11 undergraduate students, 5 PhD candidates). The highest educational level of the remaining participants was a General Certificate of Secondary Education (1) or an Advanced Level school degree (8).

Inclusion criteria for participants were: English speaking, 18 years or older and self-reported normal or corrected vision. Exclusion criteria included: self-reported history of psychiatric disorders and neurological disease, being on medication for epilepsy, migraines, renal disease, cardiac disease, hypertension, diabetes, or any other medical condition - assessed using a standard pre-screening questionnaire used at the Liverpool Magnetic Resonance Imaging Centre. All participants gave written informed consent. Part of the data was also included in a previous study (Spray et al., 2017). Ethical approval for the project was obtained from the University of Liverpool Research Ethics Committee.

### 2.2 The revised Launay-Slade Hallucination Scale (LSHS-R)

The LSHS-R (Launay and Slade, 1981) is a widely used and reliable (Bentall and Slade, 1985) self-report measure of hallucination-proneness. The 12 items describe clinical and subclinical forms of auditory and visual hallucination-like experiences. Participants are asked to rate the degree to which the content of each item applies to themselves on a 5-point Likert scale (0 = “certainly does not apply” to 4 = “certainly applies”). Aggregate scores may vary from 0 to 48, whereby high scores reflect a high degree of hallucination proneness. The LSHS-R of the current (non-clinical) sample were normally distributed with a mean score of 13.6 (±9.1) and had excellent internal consistency (α = 0.91). The mean of the current sample did not significantly differ from the mean of a larger sample from the same target population (Berry et al., 2018).

### 2.3 Data acquisition

MRI data were acquired using a 3 Tesla Siemens Trio MR scanner (Erlangen, Germany). Participants lay supine (head first) in the scanner with cushions used to minimise movement. One high-resolution T1-weighted structural run, one T2-weighted structural run and one diffusion-weighted run were acquired for each scan. The anatomical T1-weighted images (repetition time: 2040 ms, echo time: 5.57 ms, flip angle: 90°, voxel size: 1 × 1 × 1 mm^3^, field of view: 256 × 224 mm^2^) were acquired by a magnetization-prepared rapid-acquisition gradient-echo) sequence across 176 sagittal slices covering the whole brain. The structural T2-weighted images were acquired as part of the routine protocol, but not analysed further. Diffusion-weighted images (DWI) were acquired by a single shot pulsed gradient echo sequence with echo-planar read-out across 40 axial slices (repetition time: 6000 ms, echo time: 112 ms, flip angle: 90°, 3 × 3 × 3 mm^3^, field of view: 222 × 222 mm^2^). Diffusion was acquired along 60 equally distributed orientations with b-values of 1000 s/mm^2^ and 2000 s/mm^2^ with b-zero interspersed into the acquisition sequence.

### 2.4 Cortical reconstruction

T1-weighted structural images of each individual brain were automatically reconstructed by Freesurfer (Martinos Center for Biomedical Imaging, Charlestown, MA). This reconstruction automatically segmented brain images into cortical grey matter and subcortical white matter structures (Fischl, 2012). As part of this processing pipeline Freesurfer automatically computes volumetric statistics for each subject across a default set of cortical regions.

### 2.5 Definition of cortical regions of interest (ROI)

Previous studies (Allen et al., 2008; Allen et al., 2012; Van Os et al., 2009) implicate the STG in the aetiology of AVHs. We therefore assessed this region of interest (ROI) in the left and right hemispheres. The ROI was identified in each individual brain using the automatic cortical segmentation from the Freesurfer reconstruction. NODDI metrics were quantified in this ROI bilaterally for each individual using an in-house script written on MATAB 2015a (MathWorks, Natick, US).

### 2.6 Diffusion-weighted image processing

Diffusion weighted images were processed off-line using the FMRIB's Diffusion Toolbox (FDT) provided by FSL (The Oxford Centre for Functional Magnetic Resonance Imaging of the Brain) (Jenkinson et al., 2012). Pre-processing included eddy current correction and a motion correction to compensate for head motion artefacts.

For each individual, the diffusion-weighted images were linearly registered to the reconstructed anatomical space using the FLIRT tool provided by FSL. The registration matrices were produced using six degrees of freedom and were visually inspected and manually corrected if necessary.

The diffusion tensor model (Basser et al., 1994) was fitted to each voxel of the preprocessed DWI images (with b-value = 1000 s/mm^2^ and b-zero) by the DTIfit tool of FSL. Subsequently, the fractional anisotropy (FA) and mean diffusivity (MD) (Basser et al., 1994) were calculated. FA expresses the degree of anisotropic diffusion (ranging from 0 = isotropic to 1 = anisotropic) by the normalized variance of the eigenvalues of the tensor model. MD expresses the average degree of diffusion – calculated as the mean of the three eigenvalues.

The NODDI microstructure parameter maps were estimated using motion-corrected images using the NODDI toolbox(Zhang et al., 2012). The two (unitless) parameters of interest from the NODDI model were the intra-cellular volume fraction, which reflects a neurite density index (NDI), and the orientation dispersion index (ODI). The NDI expresses the fraction of diffusion per voxel within neurites and theoretically ranges from 0 (no intra-neurite diffusion) to 1 (full intra-neurite diffusion). The ODI is a measure of the dispersion of neurites (axons, dendrites) ranging from 0 (strictly parallel) to 1 (isotropically dispersed).

### 2.7 Statistical analysis

For each hemisphere (left and right STG), a multiple regression was run between all MRI metrics (FA, MD, ODI, NDI and volume) and the LSHS score. It is well known that NODDI metrics provide sensitive correlates of age (Chang et al., 2015). We therefore controlled for participant's age using this as an additional regressor of no interest. Education level may also affect cognitive performance and cerebral microstructure (Piras et al., 2011). Therefore, education level (coded by highest degree: 1 = General Certificate, 2 = Advanced Level, 3 = undergraduate studies, 4 = post-graduate studies) was added as regressor. As the full regression model (based on all measures) assesses the contribution of each measure only within the context of the other measures, subsequent partial correlations (tested by two-tailed *t*-tests) for single measures (controlling for age and education level) were examined.

## 3 Results

A multiple regression including all brain measures of the left STG (volume, FA, MD, ODI, MDI) as well as age and education level explained R^2^ = 64.8% (*R* = 0.805) of the variance in LSHS scores, adjusted R^2^ = 0.504, *F*(7, 17) = 4.48, *p* = .005. However, only volume (*β* = −0.34, *p* = .042), FA (*β* = −0.56, *p* = .003), and ODI (*β* = −0.58, *p* = .003) were significant predictors for the LSHS scores. Neither MD (*β* = −0.25, *p* = .164) and NDI (*β* = −0.07, *p* = .722) nor age (*β* = −0.16, *p* = .407) and education level (*β* = 0.12, *p* = .592) contributed significantly to the prediction of LSHS scores. A multiple regression for measures of the right STG explained only R^2^ = 16.6% (*R* = 0.408) of the variance in LSHS scores, which was not significant, adjusted R^2^ = −0.177, *F*(7, 17) = 0.48, *p* = .833.

**Fig. 1:**
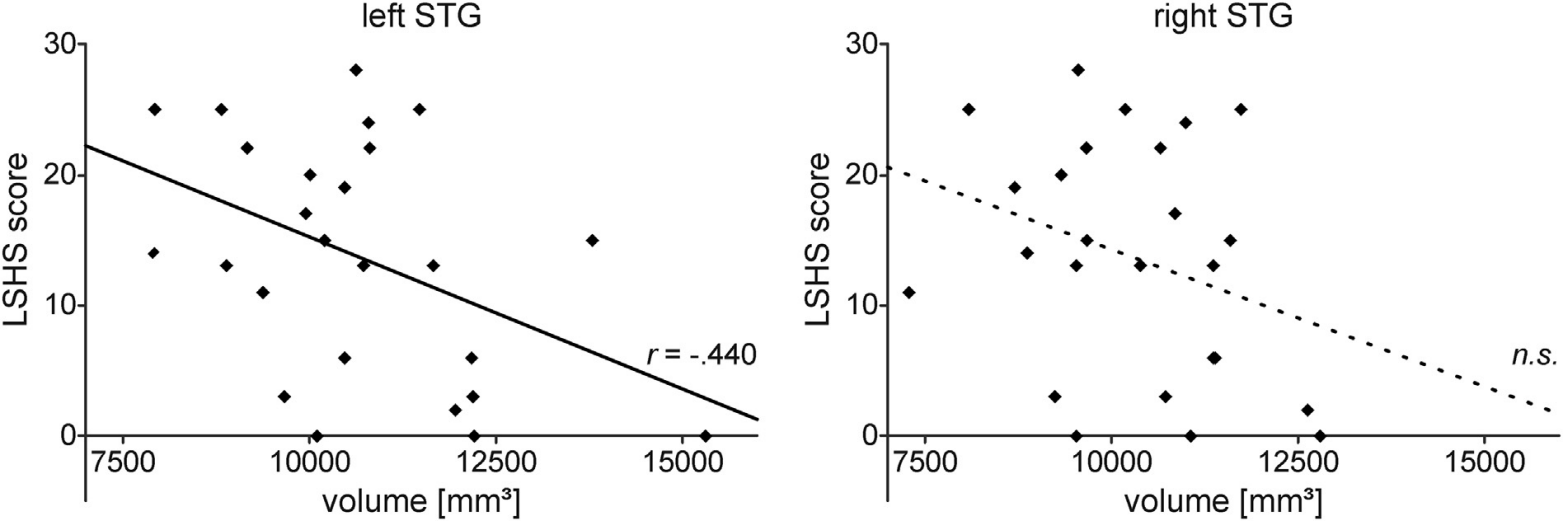
Relationship between STG volume and hallucination proneness. The scatter plots show scores of hallucination proneness (LSHS) as a function of the left and right superior temporal gyrus (STG) volume, respectively. The volume of the left STG (but not the right STG) showed a significant correlation with LSHS scores. Relationships that were significant (*p* < .05) in the multiple regression (see text) are indicated by sold lines and the partial correlation coefficient (r), non-significant (n.s.) by dashed regression lines. *n* = 25.

In order to examine the relationship between each single brain measure and hallucination proneness (LSHS) partial correlations controlling for age and education level were performed. There was a negative correlation between LSHS scores (13.6 ± 9.1) and the left STG volume (10,677 ± 1705 mm^3^), which was statistically significant, *r*(21) = −0.440, *p* = .036. However, the right STG volume (10,286 ± 1348 mm^3^) did not significantly correlate with LSHS scores, *r*(21) = −0.291, *p* = .178 (see Fig. 1).

**Fig. 2:**
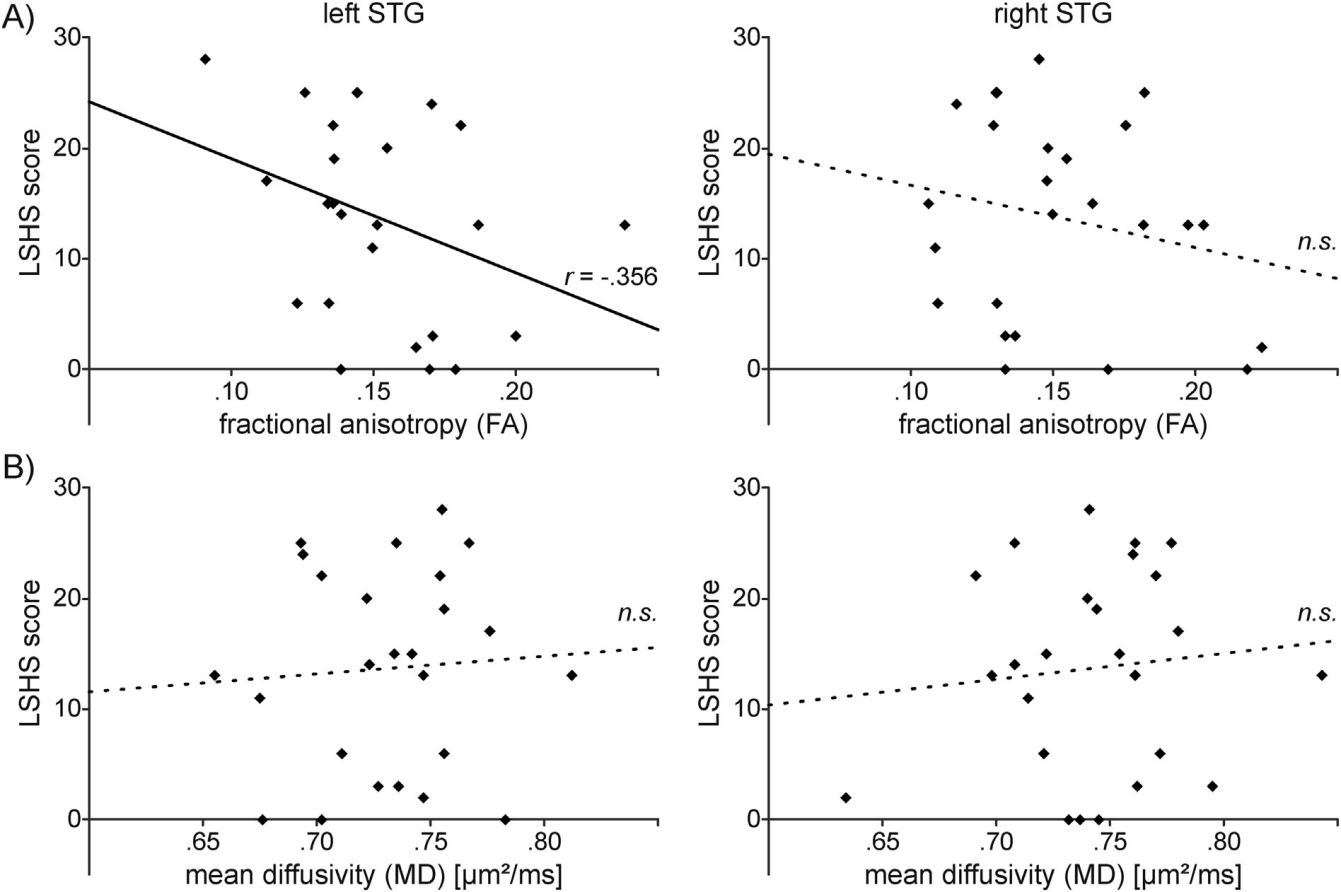
Relationship between DTI-based measures of microstructure and hallucination proneness. The scatter plots show scores of hallucination proneness (LSHS) as a function of A) fractional anisotropy (FA) and B) mean diffusivity (MD) of the left and right superior temporal gyrus (STG), respectively. FA values (but not MD) in the left STG (but not the right STG) showed a significant correlation with LSHS scores. Relationships that were significant (p < .05) in the multiple regression (see text) are indicated by sold lines and the partial correlation coefficient (r), non-significant (n.s.) by dashed regression lines. n = 25.

Partial correlations examining the relationship between hallucination proneness (LSHS) and DTI-based measures of the STG microstructure (controlling for age and education level) showed only a marginally significant negative correlation between LSHS scores and FA values in the left STG (0.15 ± 0.03), *r*(21) = −0.356, *p* = .095. FA values in the right STG (0.15 ± 0.03) were not correlated with LSHS scores, *r*(21) = −0.215, *p* = .324. MD values in the left STG (0.73 ± 0.04 μm^2^/ms) were not correlated with LSHS scores, *r*(21) = 0.033, *p* = .881, nor were MD values in the right STG (0.74 ± 0.04 μm^2^/ms), *r*(21) = 0.038, *p* = .863 (see Fig. 2).

**Fig. 3:**
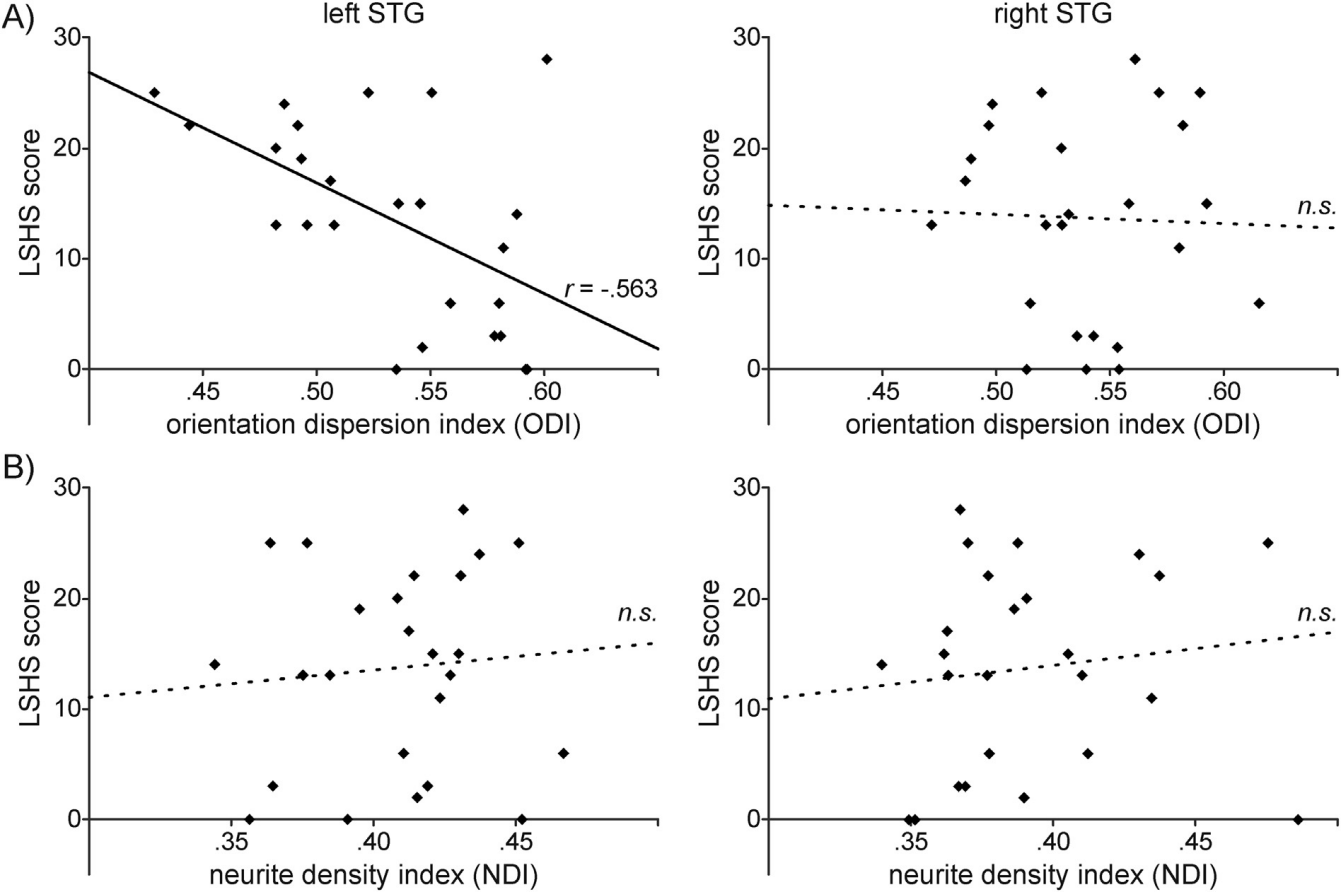
Relationship between NODDI-based measures of microstructure and hallucination proneness. The scatter plots show scores of hallucination proneness (LSHS) as a function of A) orientation dispersion (ODI) and B) neurite density (NDI) of the left and right superior temporal gyrus (STG), respectively. ODI values (but not NDI) in the left STG (but not the right STG) showed a significant correlation with LSHS scores. Relationships that were significant (p < .05) in the multiple regression (see text) are indicated by sold lines and the partial correlation coefficient (r), non-significant (n.s.) by dashed regression lines. n = 25.

Finally, we examined the relationship between hallucination proneness and neurite dispersion (ODI) and neurite density (NDI) based on the NODDI model. There was a strong, negative correlation between LSHS scores and ODI values in the left STG (0.53 ± 0.05), which was statistically significant, *r*(21) = −0.563, *p* = .005. ODI values in the right STG (0.54 ± 0.04) were not significantly correlated with LSHS scores, *r*(21) = 0.041, *p* = .851. This relationship is illustrated in Fig. 3A. Moreover, NDI values in the left STG (0.41 ± 0.03) were not correlated with LSHS scores, *r*(21) = 0.101, *p* = .645, nor were NDI values in the right STG (0.39 ± 0.04), *r*(21) = 0.146, *p* = .505 (Fig. 3B).

## 4 Discussion

Hallucination proneness (assessed using the LSHS) was shown to be negatively associated with the volume and microstructure (assessed with FA and ODI) in the left but not the right STG. Particularly, neurite complexity (ODI) rather than neurite density (NDI) was shown to be associated with hallucination proneness. These results support the proposed link between STG volume and AVH (Allen et al., 2008; Allen et al., 2012; Jardri et al., 2012). They are also consistent with the continuum model of AVHs (Baumeister et al., 2017; Van Os et al., 2009; Verdoux and van Os, 2002), demonstrating that, in a healthy population, individual variations in hallucination proneness may be related to individual differences in left STG neurite configuration.

Our results showed that several measures of brain structure (volume, FA, and ODI) in the left STG predicted LSHS scores. The strongest relationship (e.g., largest effect as indicated by correlation coefficients) was observed for ODI suggesting that NODDI-based metrics are most sensitive in detecting the relationship between brain structure and hallucination proneness. Hence, the results from the current study suggest that future research aiming to investigate links between STG structure and AVHs should adopt multi-shell diffusion-weighted MRI combined with biophysical modelling. Nevertheless, the other measures (volume, FA) still showed a relationship with hallucination proneness even when analysed together with ODI in a combined regression model. In fact, brain volume and FA were not or only weakly correlated with ODI (all *p* > .15).

The specific patterns of association between LSHS scores and the NODDI metrics provides further insight into the type of microstructural tissue configuration that may be involved in hallucination proneness and expands on previous research linking hallucinations and microstructure in the left STG (Lee et al., 2009). Healthy grey matter which is involved in higher order processing is associated with high dendritic spine complexity (Hering and Morgan, 2001; Zhang et al., 2012). We observed a negative relationship between ODI in the left STG and LSHS scores such that lower dendritic spine complexity was associated with higher hallucination proneness. As we did not find an association with hallucination proneness and dendritic density, our results support the notion that function can be regulated by dendritic spine structure and not only by the density of dendrites (Hering and Morgan, 2001; Jacobs et al., 2001; Morris et al., 2016).

Previous research suggested that AVHs are related to reduced functional connectivity within language circuitries (Lawrie et al., 2002; Mechelli et al., 2007). Although this reduced functional connectivity may be mediated by the architecture of axons in the white matter (e.g., callosum)(Spray et al., 2017), the present results suggest an additional mechanism: The reduced functional integration in AVHs may reflect reduced synaptic integration capacity within the grey matter of the left STG. This reduced synaptic integration seems to be primarily due to reduced neurite complexity. However, as we also observed volume changes, which were not or only weakly correlated with ODI, other mechanisms may also contribute. Further research assessing both functional integration capacity in the left STG alongside measures of synaptic integration capacity (such as ODI) in the left STG could shed further light on the mechanisms behind AVH and offer a potential biological marker for this symptom.

The sample of the current study did not include CVHs which means that the results are not confounded by illness-related effects of chronicity and medication. Although this is an advantage, future research needs to determine whether the findings are generalizable to a clinical population. Although healthy individuals who score highly on the LSHS do not usually have hallucinatory experiences that are as pervasive and persistent as those experienced by CVHs (Stanghellini et al., 2012), HVHs with high LSHS scores and CVHs have similarly impaired source monitoring ability (Brébion et al., 2016; Brookwell et al., 2013). Moreover, previous research suggests that the AVH neural mechanisms are likely to be shared by both groups (Jardri et al., 2012). There are some indications from self-report studies that high LSHS scores in HVHs may be linked to excessive dialogic inner speech (McCarthy-Jones and Fernyhough, 2011) whereas CVHs may especially experience inner speech that takes the characteristic of other people (de Sousa et al., 2016). However, the main difference between CVHs and HVHs seems to be in the way that they interpret their experiences, with CVHs interpreting their hallucinations as powerful, threatening and therefore distressing (Chadwick and Birchwood, 1994; Daalman et al., 2011; Honig et al., 1998; Sorrell et al., 2010). Future research into the neurite configuration of the STG in groups of both HVHs and CVHs could further advance our understanding of group differences and help to determine whether the neurite related AVH aetiology is shared.

A limitation of the current study is the relatively small and homogenous (primarily students) sample. Future research may seek to replicate these findings with a larger sample from a more diverse population. Additional characteristics, not assessed in this current study, such as levels of anxiety or depression, intelligence, socioeconomic status, and history of psychiatric disease could also be associated with hallucination proneness, brain volume, and microstructure. Future research should assess the contribution of these additional factors - even though in our study age and educational level had only a very moderate effect compared to the metrics of brain structure.

In summary, the current findings point towards a possible mechanism for hallucinations within the left STG. The findings suggest that an aberrant microstructure, specifically reduced dendritic spine complexity, contributes - at least partially - to the genesis of AVHs. Furthermore, the present results suggest that multi-compartment DWI methods such as NODDI provide more sensitive measures than volumetric measures alone.
